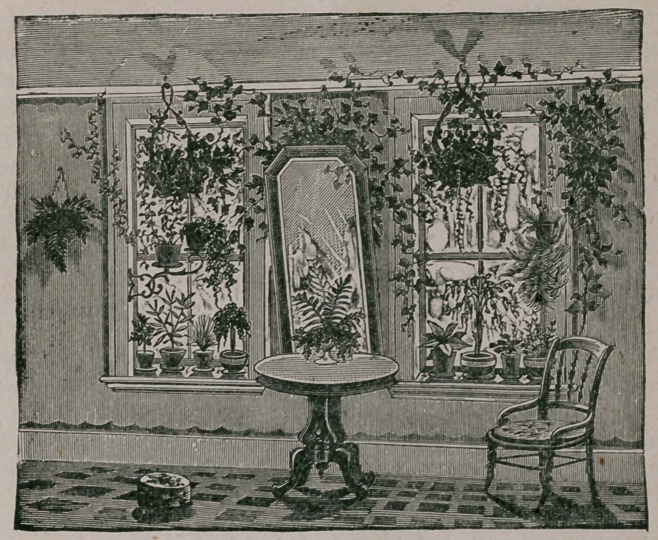# Household

**Published:** 1888-03

**Authors:** 


### HOUSEHOLD.

The above cut, the last of the series of "window gardening," presented in previous numbers of the JOURNAL, illustrates the ornamentation of ordinary single cottage windows, and will be sufficiently understood of itself.

There is no home, however humble and unpretentious, which may not be given an air of cultivation and refinement by the exercise of a little taste in the distribution and care of a few simple flowers, which always lend cheerfulness and beauty to their surroundings. But this is not all, the cultivation of flowers is, in itself, a school of refinement and good taste, which amply repays all the attention bestowed in this direction.

Distribution of plants in the house is important. In a collection we may have plants from many climates ; therefore, difficulties may be avoided by carefully distributing the plants in the various parts of the greenhouse ; not only does the temperature vary, but the light does not penetrate equally the different parts of the house. Thus, one plant may do better right up against the glass, while others prefer a half shady nook ; others, again, would do better in the warmest part, while some delight in a cool and moist end or side of the house. Yet it is rather a difficult task even for an experienced grower, to place every plant in the right place, while a beginner will seldom do the right thing. The only thing to do in this direction is to study the natural habitat of each species, and then place it in a position nearest to the description of the natural one. Plants that grow naturally upon exposed rocks and trees in the savannah of South America or jungles of India, will require a warm and sunny place, while those inhabiting the mountainous regions of high altitudes require a cool, moist and partly shaded place. Many will be found easy to grow when their wants are better known, just as much as the cool orchids which in former times were considered difficult to cultivate because they were grown in company of those requiring strong heat.

A "Water-Light."—A new principle for keeping plants through the winter without artificial heat, was recently shown at the Regent's Park, London, with the plants grown in them last winter. The essence of the invention is, that all light and heat shall previously pass through a shallow layer of water. The water is found to exercise great control over temperature, protecting plants entirely from frost in winter and from excessive direct heat in summer. The application involves no difficulty. In the case of a garden frame, a sliding "water-light" about three inches deep is made to fit over the frame containing the plants; the only difference from a glass light being, that it holds water, and is always placed in a flat position. The depth of water generally kept in the tank is about two inches in winter and summer, and half the depth in spring and autumn.

Scalloped Potatoes.—Cut up cold boiled potatoes until you have about a quart. Put in a pan a generous cup of milk, one teaspoonful flour and one tablespoonful butter. Set on the stove and let it thicken, then put a layer of potatoes in a pudding dish, season with salt and pepper, and pour on a little of the gravy. Continue until it is all used. Cover the top with rolled cracker crumbs and bits of butter. Bake twenty minutes.

To Destroy Insects.—One of the cheapest and best modes of destroying insects in pot plants is to invert the pot, and dip the plants for a few seconds in water warmed to 130°. A German paper, referring to this plan, says that the azalea will stand 133° without injury. We usually heat the water pretty well, and pour in cool until 130° is reached.

Bread Pudding.—Quart sweet milk, quart bread crumbs, four eggs, four tablespoonfuls sugar; soak bread till soft in half the milk, mash fine, add the rest of the milk, the beaten eggs, sugar, and a teacupful raisins. Bake one hour, serve warm with a warm sauce, maple sugar hard sauce, or with whipped or sweetened cream.

An appetizing way to warm over potatoes is to heat them in a saucepan containing a coffee cupful of beef stock, well seasoned with salt, pepper, and a little parsley which has first been browned in butter; cut the potatoes in thin slices and let them cook slowly until they are saturated with the gravy or liquor.

Orange Pudding.—Peel and slice half a dozen small oranges and lay in a deep dish, and scatter sugar plentifully on as if they were to be eaten raw. Make a soft custard of one pint of milk, and tablespoonful of rice flour, four tablespoonfuls of sugar (heaped), and the yolks of three eggs; cook it in a double boiler, and when it has thickened take it from the fire; flavor with lemon and pour over the oranges; put the dish in the oven and bake fifteen or twenty minutes, then draw it to the front and put a meringue over the top, made of the beaten whites of the eggs and a heaping teaspoonful of sugar.

## Figures and Tables

**Figure f1:**